# Acute Toxicity of Amorphous Silica Nanoparticles in Intravenously Exposed ICR Mice

**DOI:** 10.1371/journal.pone.0061346

**Published:** 2013-04-12

**Authors:** Yang Yu, Yang Li, Wen Wang, Minghua Jin, Zhongjun Du, Yanbo Li, Junchao Duan, Yongbo Yu, Zhiwei Sun

**Affiliations:** 1 School of Public Health, Capital Medical University, Beijing, People's Republic of China; 2 School of Public Health, Jilin University, Changchun, Jilin, People's Republic of China; Brandeis University, United States of America

## Abstract

This study aimed to evaluate the acute toxicity of intravenously administrated amorphous silica nanoparticles (SNPs) in mice. The lethal dose, 50 (LD_50_), of intravenously administrated SNPs was calculated in mice using Dixon's up-and-down method (262.45±33.78 mg/kg). The acute toxicity was evaluated at 14 d after intravenous injection of SNPs at 29.5, 103.5 and 177.5 mg/kg in mice. A silicon content analysis using ICP-OES found that SNPs mainly distributed in the resident macrophages of the liver (10.24%ID/g), spleen (34.78%ID/g) and lung (1.96%ID/g). TEM imaging showed only a small amount in the hepatocytes of the liver and in the capillary endothelial cells of the lung and kidney. The levels of serum LDH, AST and ALT were all elevated in the SNP treated groups. A histological examination showed lymphocytic infiltration, granuloma formation, and hydropic degeneration in liver hepatocytes; megakaryocyte hyperplasia in the spleen; and pneumonemia and pulmonary interstitial thickening in the lung of the SNP treated groups. A CD68 immunohistochemistry stain indicated SNPs induced macrophage proliferation in the liver and spleen. The results suggest injuries induced by the SNPs in the liver, spleen and lungs. Mononuclear phagocytic cells played an important role in the injury process.

## Introduction

Nanomaterials have been widely used in a variety of fields and the potential hazards to the environment and humans are attracting increasing attention. Amorphous silica nanoparticles (SNPs) are one of the most common nanomaterials and because of their favorable physico-chemical properties, they are being applied increasingly more in industrial manufacturing, high-molecule composite materials, cosmetics, and foodstuffs [1]. Because of their high hydrophilicity, good biocompatibility, easy surface modification and labeling, silica nanoparticles are being developed for a host of biomedical and pharmaceutical applications such as drug delivery, cancer therapy, imaging probes, biosensors and enzyme immobilization [2], [3]. Human exposure to the SNPs is increasing; therefore, the evaluation of the toxicity of these nanoparticles is urgently needed.

To date, the results of a growing number of in vitro studies have shown that the cytotoxicity induced by amorphous SNPs is dose-, time-, size- and cell line-dependent [4], [5], [6]. SNPs can enter cells through different routes and then distribute in the cytoplasm and nucleus [7], [8]. Reactive oxygen species (ROS) formation has been considered as a mechanism involved in the toxic effect of SNPs [9], [10]. As reported, SNP exposure leads to an oxidative stress and inflammation response in various cell lines followed by cell membrane damage, DNA strand breaks, mitochondrial dysfunction, cell cycle arrest, necrosis and apoptosis [6], [11], [12]. However, in vivo toxicity of SNPs has been studied far less than in vitro toxicity [3].

Inhalation is a common route for exposure to nanomaterials. Thus, much research has been performed on the pulmonary toxicity caused by amorphous SNPs. Animal inhalation studies indicate that exposure to SNPs results in transient changes in breathing parameters, increased lung weight, total bronchoalveolar lavage (BAL) cells and proteins, induced acute inflammation and tissue damage [13], [14], [15].

In recent years, because of the application of SNPs to biomedicine and biotechnology, intravenous exposure to SNPs has become common, but little research has been carried out to assess the toxicity of intravenous SNP exposure [16], [17], [18] and there is still no uniform standard for determining the toxicity of nanomaterials entering the blood stream [19]. The acute toxicity studies that have been done on intravenous SNP exposure are limited and far from comprehensive. Additionally, there is no available LD_50_ for SNPs for toxicity grading. Acute toxicity research is the first step to understand the toxic effects of chemicals on organisms, to provide a basis for subsequent subchronic and chronic toxicity study. Thus, it has become important to clearly indentify the acute toxicity of SNPs.

In the present study, we systematically evaluated the acute toxicity in mice of intravenously injected 64 nm SNPs in order to provide experimental evidence for the evaluation of the toxicity of silica nanomaterials. The LD_50_ of SNPs in ICR mice was estimated for the first time using Dixon's up-and-down method. Dead animals from this experiment were sent for an immediate necropsy to identify the cause of death. Then, for acute toxicity research, a series of doses were set based on the LD_50_. The silicon content of tissues was determined by an inductively coupled plasma-optical emission spectrometer (ICP-OES). Blood biochemical assay, morphological and histopathological examination and TEM imaging were used to investigate the adverse effects of SNPs on major organs.

## Materials and Methods

### Silica nanoparticles

The amorphous SNPs (12 g/L mass concentration) were provided by the school of chemistry at Jilin University. The shape and average size of the particles were measured by transmission electron microscope (TEM) (JEOL, Japan). A Zeta electric potential granulometer (Malvern, Britain) was employed to examine the Zeta potential and hydrodynamic sizes of silica particles in dispersion media.

### Detection of endotoxin

Gel clot Limulus Amebocyte Lysate (LAL) assay was used to detect the endotoxin in SNP suspensions at concentrations of 0.75, 1.5, 3, 6, and 12 mg/ml. The gel clot LAL reagents including endotoxin standard, LAL water, and lysate were purchased from Zhan Jiang Bokang Marine Biological Co., LTD. The detection limit was less than 0.125 EU/ml.

### Animals

Male and female ICR mice (8 weeks old and 20–22 g in weight) were purchased from Weitong-Lihua Experimental Animal Center (Beijing, China). They were separated by sex in plastic cages with stainless steel mesh lids in a ventilated room. The room was maintained at 20±2 °C and 60±10% relative humidity with a 12 h light-dark cycle. The mice were given water and sterilized food. Prior to treatment, the mice were not fed overnight. All animal care and experimentations were approved by the Animal Ethics Committee at Capital Medical University (approval number 2011-X-072).

### LD_50_ Estimation using Dixon's Up-and-Down Method

LD_50_ was estimated using the up-and-down method [20] as described by Dixon, which uses an iterative dose-selection algorithm. The maximum likelihood estimate for LD_50_ with SE was calculated using the following formula: LD_50_ = average (Xi)+d/N×(A+C). Average (Xi) is the average experimental dose for the last N samples, N is the nominal number of samples or total number of samples, minus 1 less than the number of identical samples at the beginning of the trial, A and C values are acquired from Dixon's tables after the series of experiments are performed, and d is the distance between data points [21]. The method assumes that the SD, σ, is equal to the spacing distance. However, Dixon also gives a method to calculate SE = σ×square root of (2/N). Mortality in this LD_50_ estimating study was recorded and the bodies were sent for an immediate necropsy.

### Silicon content and tissue distribution

To determine the silicon content in the tissues, samples from the heart, liver, spleen, lung, kidney and brain were collected. The wet samples were weighed and digested with nitric acid by microwave heating, and then the silicon content was analyzed using ICP-OES (Optima 7000DV, PerkinElmer, US).

### Acute toxicity

For the SNP acute toxicity study a series of doses were set based on an LD_50_ estimating study. A total of 40 mice of either sex were exposed to 0, 29.5, 103.5 and 177.5 mg/kg of SNPs. An SNP suspension in physiological saline was injected through the mouse tail vein. Injections of sterile physiological saline were also given to the mice as a control. After the injection, symptoms and mortality were observed and carefully recorded throughout the entire experiment. At the end of the experiment, all animals were sacrificed for subsequent experimental study.

### Body weight and coefficients of organs

After exposure to the different doses of SNPs, the mice were weighed on days 1 and 14. On day 14 after the injection, the mice were sacrificed and the heart, lung, liver, spleen, kidneys and brain were excised and accurately weighed. The coefficients of these organs to body weight were calculated as the ratio of tissues (wet weight) to body weight.

### Blood biochemical assay

Blood samples were collected via the ocular vein. The serum was obtained by centrifugation of the whole blood at 3,000 rpm for 15 min. Liver function was evaluated based on the serum levels of aspartate minotransferase (AST), alanine aminotransferase (ALT) and Albumin (ALB). Nephrotoxicity was reflected by blood urea nitrogen (BUN) and creatinine (Cr). The enzyme of lactate dehydrogenase (LDH) was measured to evaluate cell membrane injury and tissue damage. These biochemical parameters were determined by an automated biochemical analyzer (Type 7200-202, Hitachi, Japan).

### Histopathological Examination

The liver, spleen, kidney, heart, lung and brain were removed and fixed in 10% formalin, embedded in paraffin, sectioned, and stained with hematoxylin and eosin (HE) for histological examination using standard techniques. In order to confirm the cause of death, the sections of the lungs in the dead mice were stained for fibrin with Martius Scarlet Blue (MSB) according to routine procedures. After staining, the slides were observed and examined by optical microscope (Olympus X71-F22PH, Japan). The pathologist was blind to the identity and analysis of the pathology slides. For quantification, the total number and size of granulomas in 30 optical fields (200× magnification) of each liver section, and the number of megakaryocytes in 50 optical fields (400× magnification) of each spleen section were determined using an Olympus software (cellSens Standard). The fields were chosen randomly and continuously. Data were presented as mean ± SD of five mice of each group.

### Immunohistochemistry

CD68 was detected immunohistochemically in the paraffin embedded liver, spleen and lung sections as a marker of macrophages. After deparaffinisation and rehydration, the sections were placed in a 10 mM citrate buffer solution (pH 6.0) for antigen retrieval. In order to quench endogenous peroxidase activity, the sections were treated with 3% H_2_O_2_ in PBS for 5 min and then washed in PBS. Then the sections were blocked with 10% normal goat serum for 10 min at 37°C and incubated overnight at 4°C with primary antibody or an equivalent amount of normal goat IgG as a negative control. The sections were then treated with an avidin-biotin affinity system for 30 min at room temperature, washed, stained with 3-3' diaminobenzidine substrate, counter stained with hematoxylin and examined under a light microscope. Activated macrophage marker CD68 was quantified as positively stained cells per high-power field. Results were expressed as the total positive numbers from 50 random and continuous fields from each section (400× magnification).

### TEM imaging

For electron microscopy, the heart, lung, liver, spleen, kidney and brain were excised and immediately fixed overnight in 3% glutaraldehyde. Then the samples were rinsed three times with 0.1 M PB and postfixed with 1% osmic acid for 2 hours. After being rinsed three times with 0.1 M PB and serially dehydrated with 50%, 70%, 80%, 90% and 100% alcohol and 100% acetone, the samples were embedded in epoxy resin for making the blocks of tissues. The ultrathin sections (50 nm) were obtained by an ultramicrotome (Ultracut UCT, Leica, Germany). They were then stained with lead citrate and uranyl acetate, and then viewed on a TEM (JEM2100, JEOL, Japan). The pathologist was blind to the identity and analysis of the ultrathin sections.

### Statistical Analysis

Statistical analysis was done with SPSS (version 16.0). The average level of variables was presented as mean ± standard deviation (SD) for continuous variables with normal or near normal distribution. Then, parametric comparisons of continuous variables were done with one-way analysis of variance (ANOVA) or analysis of covariance (ANCOVA), where appropriate. Subsequently, preplanned pairwise comparison between each experimental group and control group was done. All significance tests were two-sided at level of 0.05.

## Results

### Characterization of SNPs

**Figure 1:**
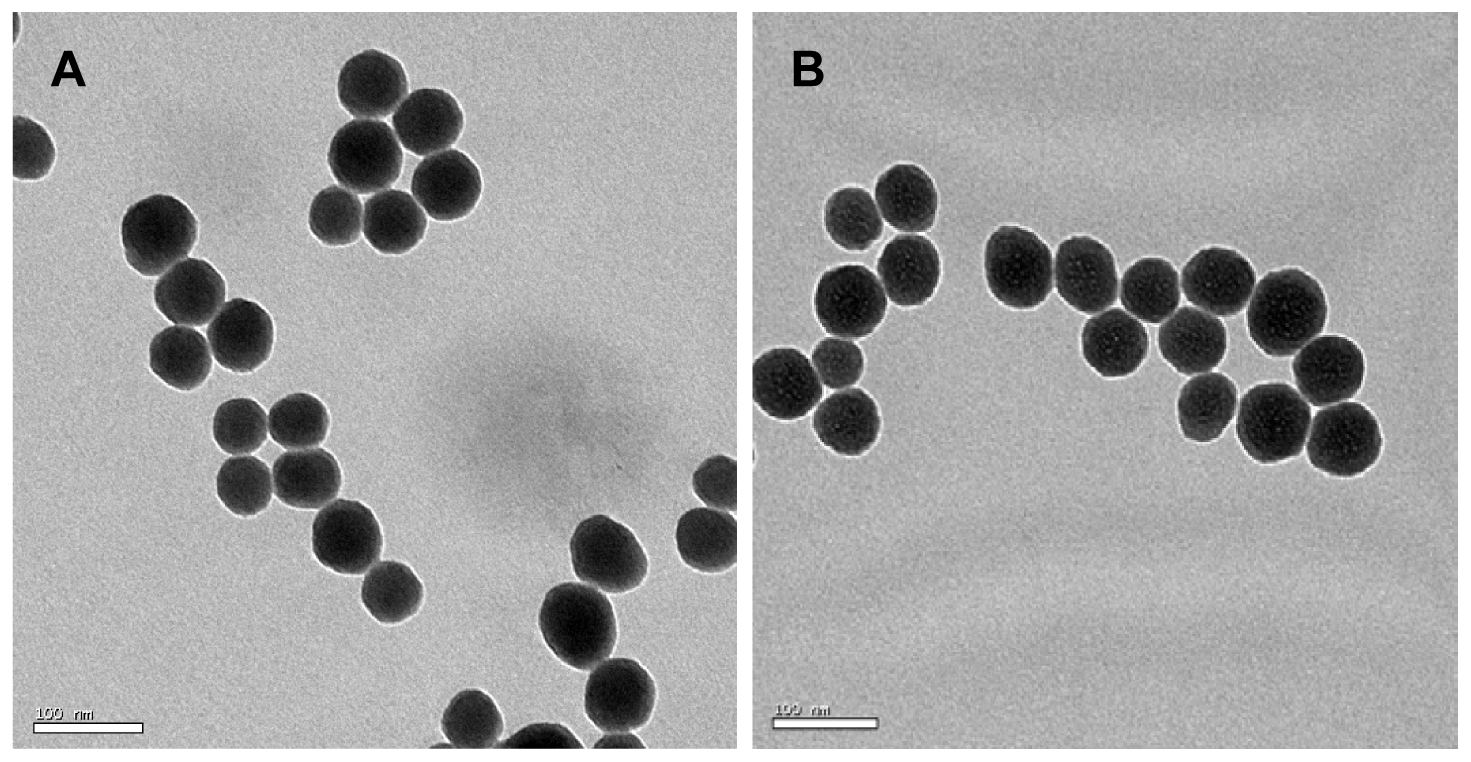
TEM images of the amorphous SNPs in distilled water or physiological saline as the dispersion media. Spherical SNPs show good monodispersity in distilled water (A) and physiological saline (B).

**Figure 2:**
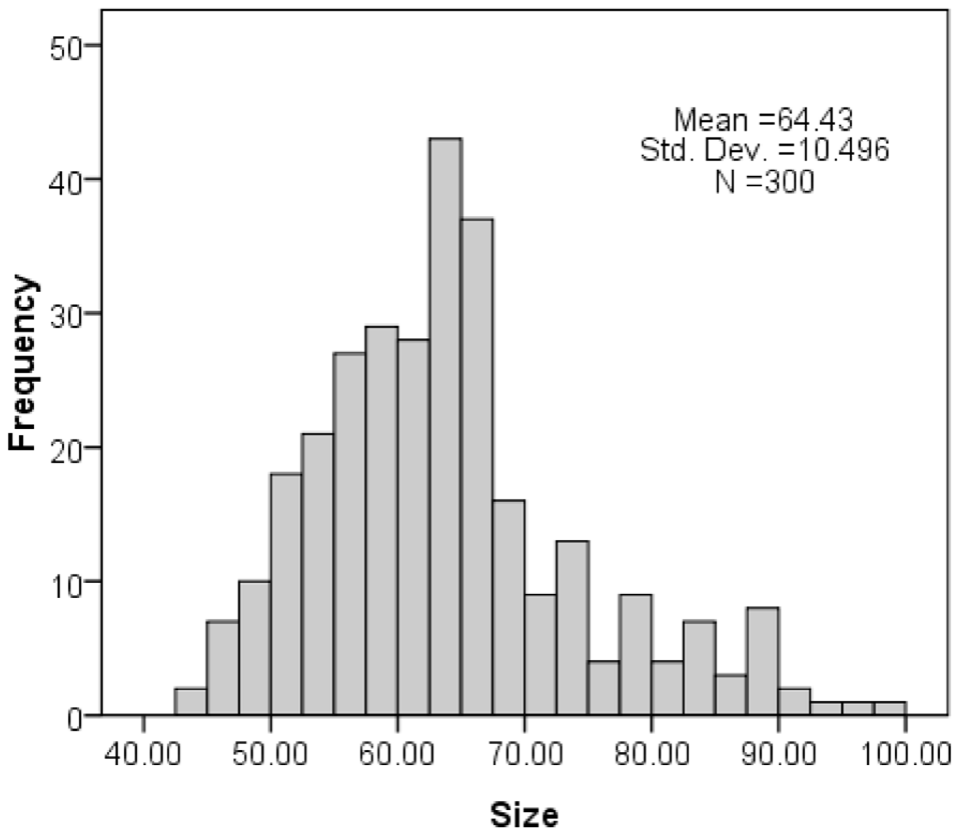
Size distribution of the amorphous SNPs.

TEM and a Zeta electric potential granulometer were used to characterize the amorphous SNPs. The average size of the spherical SNPs was 64.43±10.50 nm (Figure 1 and 2). The zeta potential and hydrodynamic size of the 64 nm SNPs were measured in the distilled water or physiological saline as the dispersion medium (Table 1). The SNPs had good monodispersability in distilled water (stock media) and in physiological saline (exposure media) with time (Figure 1, Table 1). The LAL assay indicated no detectable gram negative endotoxin on the SNPs at concentrations of 0.75, 1.5, 3, 6, and 12 mg/ml.

**Table 1:** Zeta potential and hydrodynamic size of the 64 nm SNPs in distilled water or physiological saline as the dispersion media.

	In distilled water			In physiological saline		
Time	Zeta potential (mV)	Hydrodynamic size (nm)	PDI	Zeta potential (mV)	Hydrodynamic size (nm)	PDI
10 min	−42.7	108.3	0.116	−39.2	110.4	0.119
1 h	−43.6	107.5	0.112	−41.3	111.8	0.127
6 h	−46.1	110	0.098	−40.7	108.1	0.142
12 h	−45.8	109.1	0.084	−38.3	108.8	0.141
24 h	−43.2	107.3	0.109	−43.8	107.8	0.136

### LD_50_

**Figure 3:**
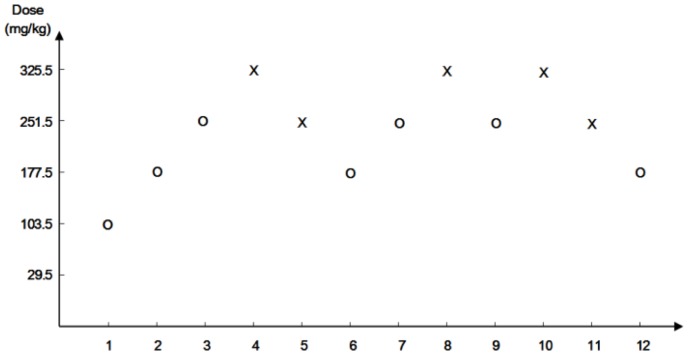
Up-and-down experimental series. The test was started with an initial dose of 103.5 mg/kg, and an interval of doses of 74 mg/kg. O = survival to 48 h; X = death within 48 h.

To obtain the LD_50_ of 64 nm SNPs in ICR mice, doses and intervals were designed according to the Dixon up-and-down method. After acclimatization to the environment, the mice were exposed to the SNPs with the doses shown in Figure 3. The first animal received a dose one step below the assumed estimate of the LD_50_. If the animal survived, the second animal received a higher dose. If the first animal died, the second animal received a lower dose. Each subsequent dosage was raised or lowered based on the survival of the preceding animal. The mortality in each dose group was observed and recorded. The LD_50_ of 64 nm SNPs was calculated using the formula provided by Dixon's up-and-down method (LD_50_ = 262.45±33.78 mg/kg).

**Figure 4:**
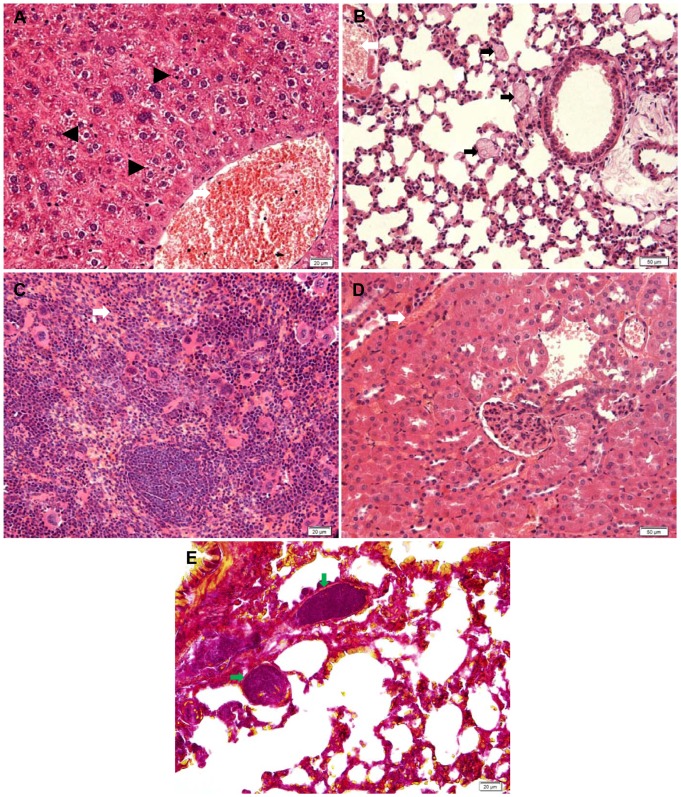
Histological analysis of the main organs in the dead mice. Representative pictures from HE staining sections of the liver (A), lung (B), spleen (C), and kidney (D) and MSB staining sections of the lungs (E) in dead mice. *Black triangles* denote serious hepatic necrosis in the liver. *Black arrows* denote thrombus probably induced by SNPs, and *white arrows* denote hyperaemia in tissues. *Green arrows* denote the red fibrin thrombi in the lungs. The magnification was 400× for A, C and E, 200×for B and D. Data are representative of 5 mice.

In this experiment, the symptoms and mortality of the treated mice were observed and recorded. Dead animals were sent for an immediate necropsy. As shown in Figure 3, three mice were injected with the SNPs at 325.5 mg/kg, and they all died in 4–5 hours after injection; five mice were injected with the SNPs at 251.5 mg/kg, two of them died in 8 hours after administration. The mice showed obvious symptoms of labored breathing, tremor, arching, cyanosis, hypothermia, difficulty in movement and sleepiness. In the gross anatomical examination, the liver, spleen, lung and kidney showed obvious swelling and hyperemia, and the capillary networks of the liver and spleen were clearly visible. Serious hepatic necrosis, remarkable central vein dilatation and congestion in the liver and pink microthrombi widely distributed in the pulmonary arterioles were observed in the pathological examination of the dead mice (Figure 4A and 4B). Megakaryocyte accumulation, increased size of red pulp in the spleen, and renal interstitium hyperemia was demonstrated in the pathological examination (Figure 4C and 4D). The red homogeneity fibrin thrombi were observed in the pulmonary arterioles in the sections of the lungs by the MSB staining (Figure 4E).

### Silicon content and tissue distribution

**Figure 5:**
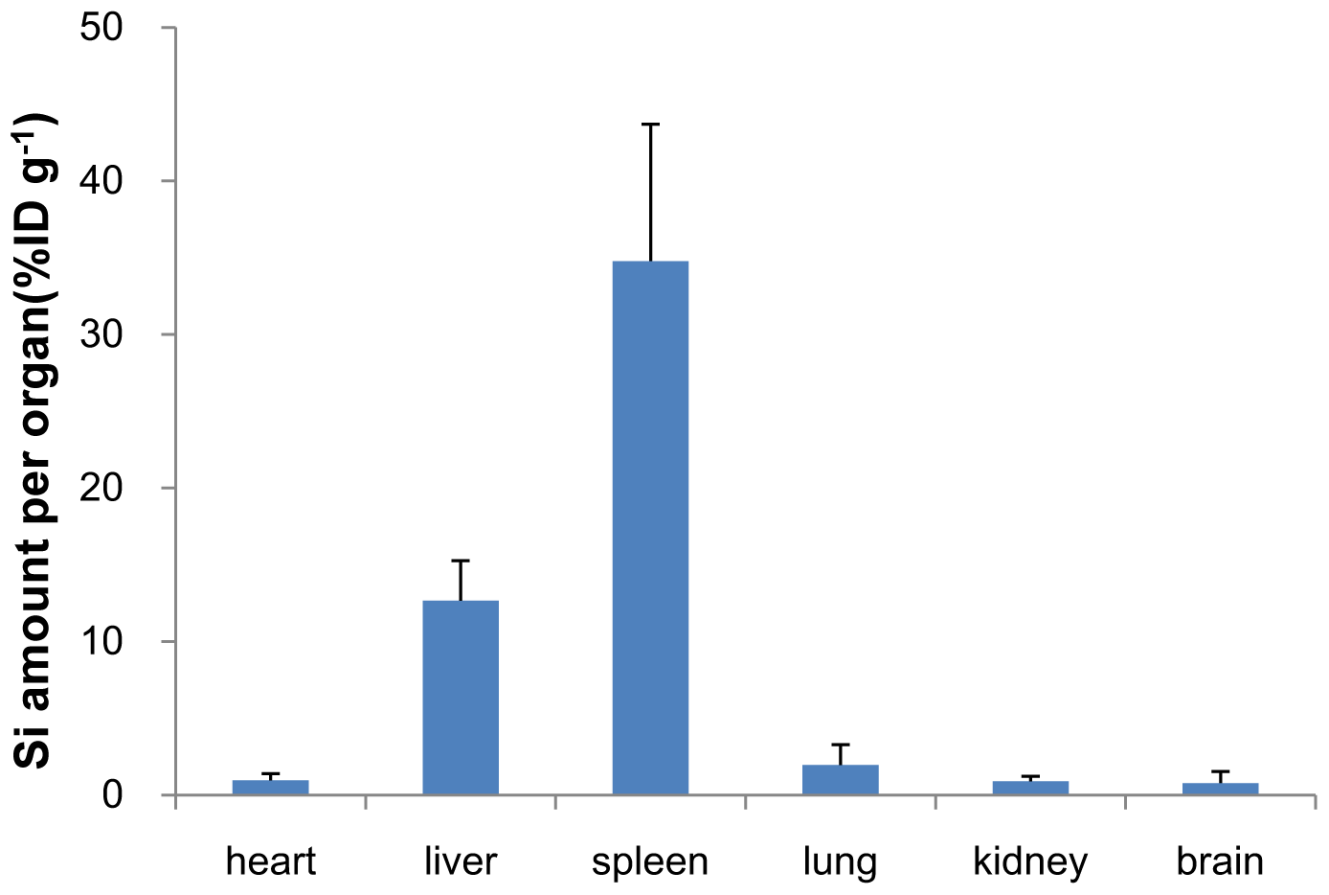
ICP-OES analysis result of the silicon levels. The silicon content was analyzed in the heart, liver, spleen, lung, kidney and brain in the SNP treated mice at 177.5 mg/kg. Data are expressed as mean ± SD (n = 9).

An analysis of the silicon content in different viscera in the SNP treated mice at 177.5 mg/kg by ICP-OES revealed that the silicon content varied in different tissues. SNPs mainly distributed or accumulated in the tissues enriched with the monocyte phagocyte system (MPS), like the liver and spleen, but not in the lung. At 14 days following the injection, about 12.67% of the injected SNPs localized in the liver and 34.78% in the spleen, while only 1.96% localized in the lung. Very little silicon was detected in the heart, kidney and brain (Figure 5). About 52.04% of the injected SNPs still remained in the body at day 14, possibly requiring a longer period for complete clearance.

### Acute toxicity study

#### Vital signs

**Figure 6:**
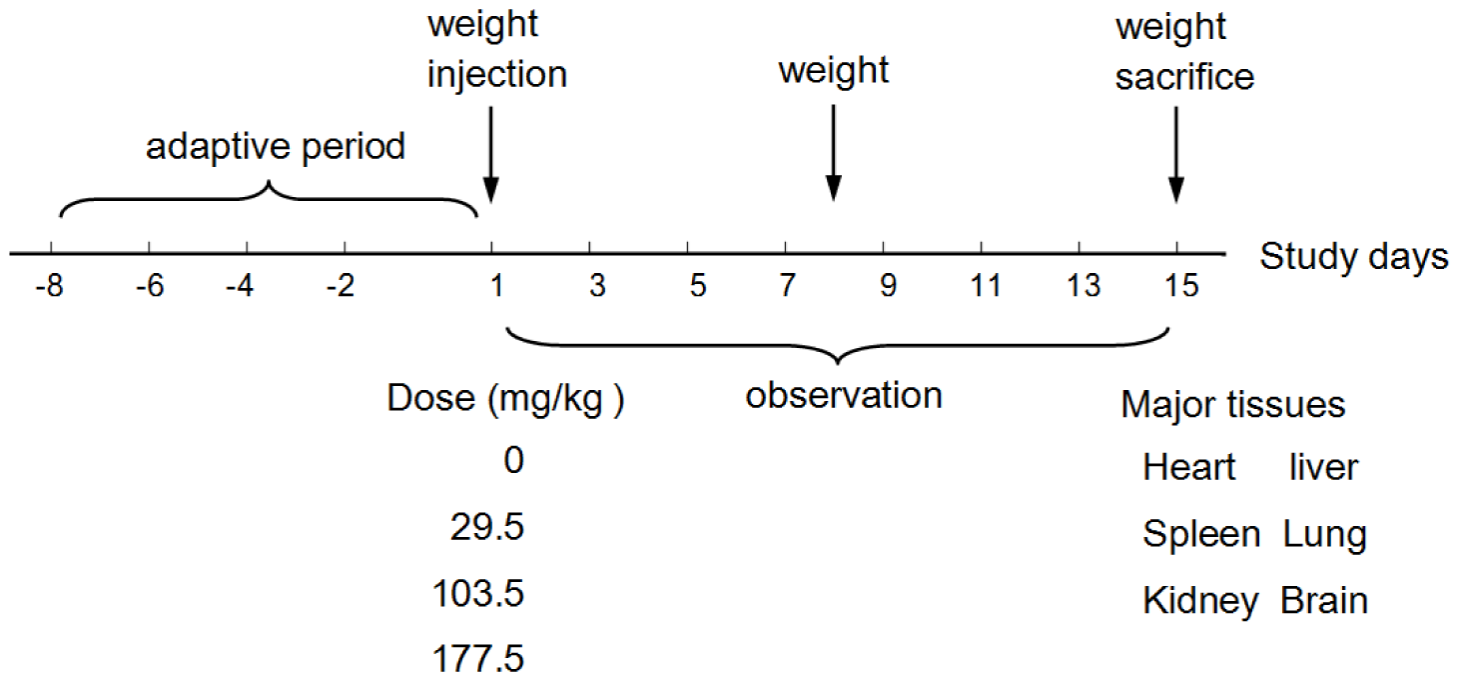
Experimental design of acute toxicity of the SNPs in ICR mice.

The experimental protocol for the acute toxicity study is shown in Figure 6. During the entire study period, no unusual behavior was observed in the control and 29.5 mg/kg treated groups. A few animals showed temporary labored breathing and cyanosis in the 103.5 mg/kg treated group. In the 177.5 mg/kg treated group, one male animal died at 6 h after injection. During the observation period, labored breathing, cyanosis and difficulty in movement were observed. Interestingly, seven mice in the 177.5 mg/kg treated group developed progressive tail ischemic necrosis and at day 14 only 2–3 cm of the tail remained. In the 103.5 mg/kg treated group, only two mice developed a slight tail ischemic necrosis (about 0.5 cm). No changes were observed in the control and 29.5 mg/kg treated groups.

#### Body weight and coefficients of organs

**Figure 7:**
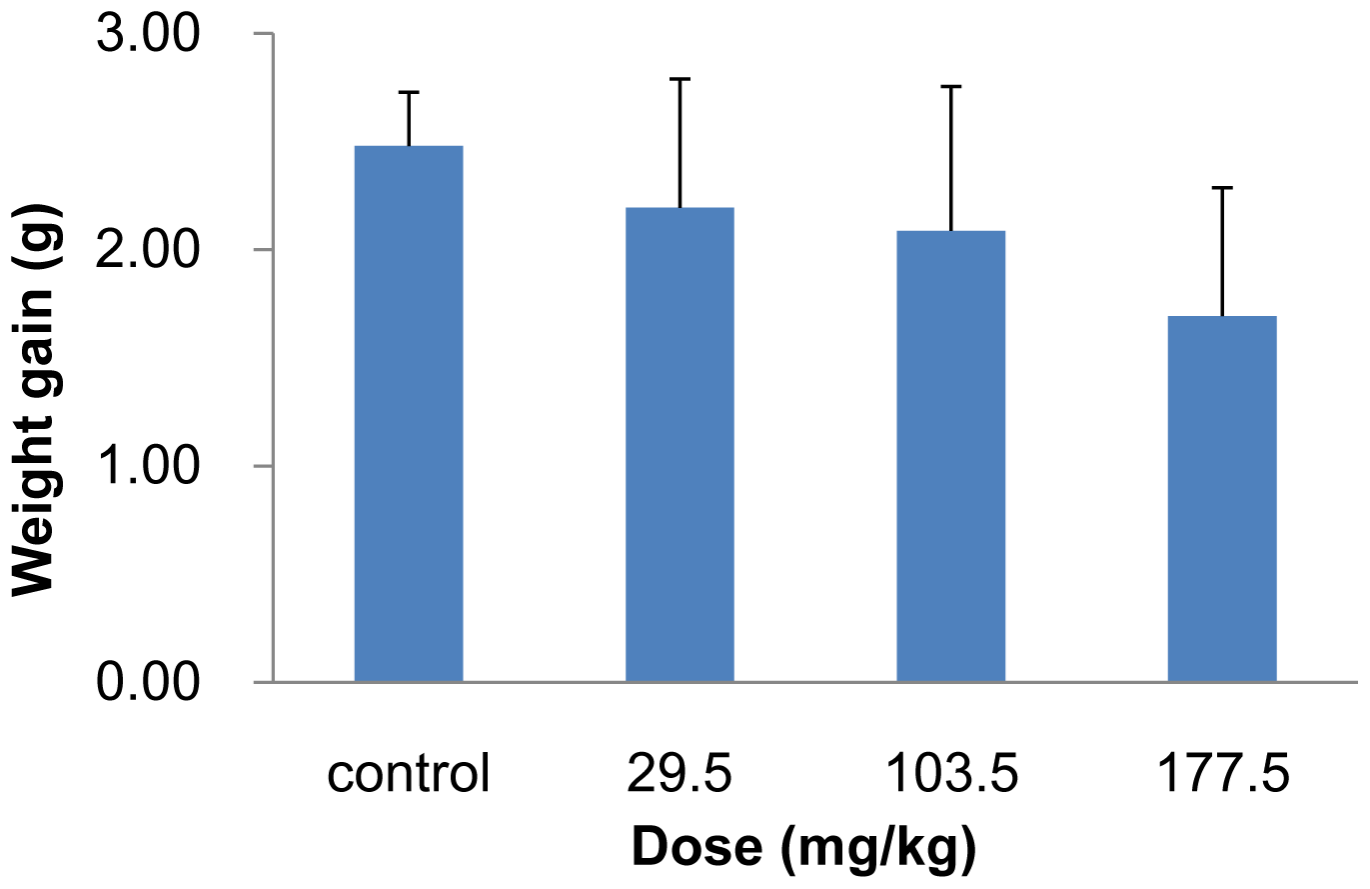
Weight gain of mice treated with the SNPs. The SNPs were intravenously administered to mice at 29.5, 103.5 and 177.5 mg/kg. Mice were weighed on days 1 and 14 after injection. The weight gain decreased in a dose-dependent manner in SNP treated mice, but with no significant difference compared with control group. Data are expressed as mean ± SD (n = 9 or 10). ANCOVA was used for the data analysis.

**Figure 8:**
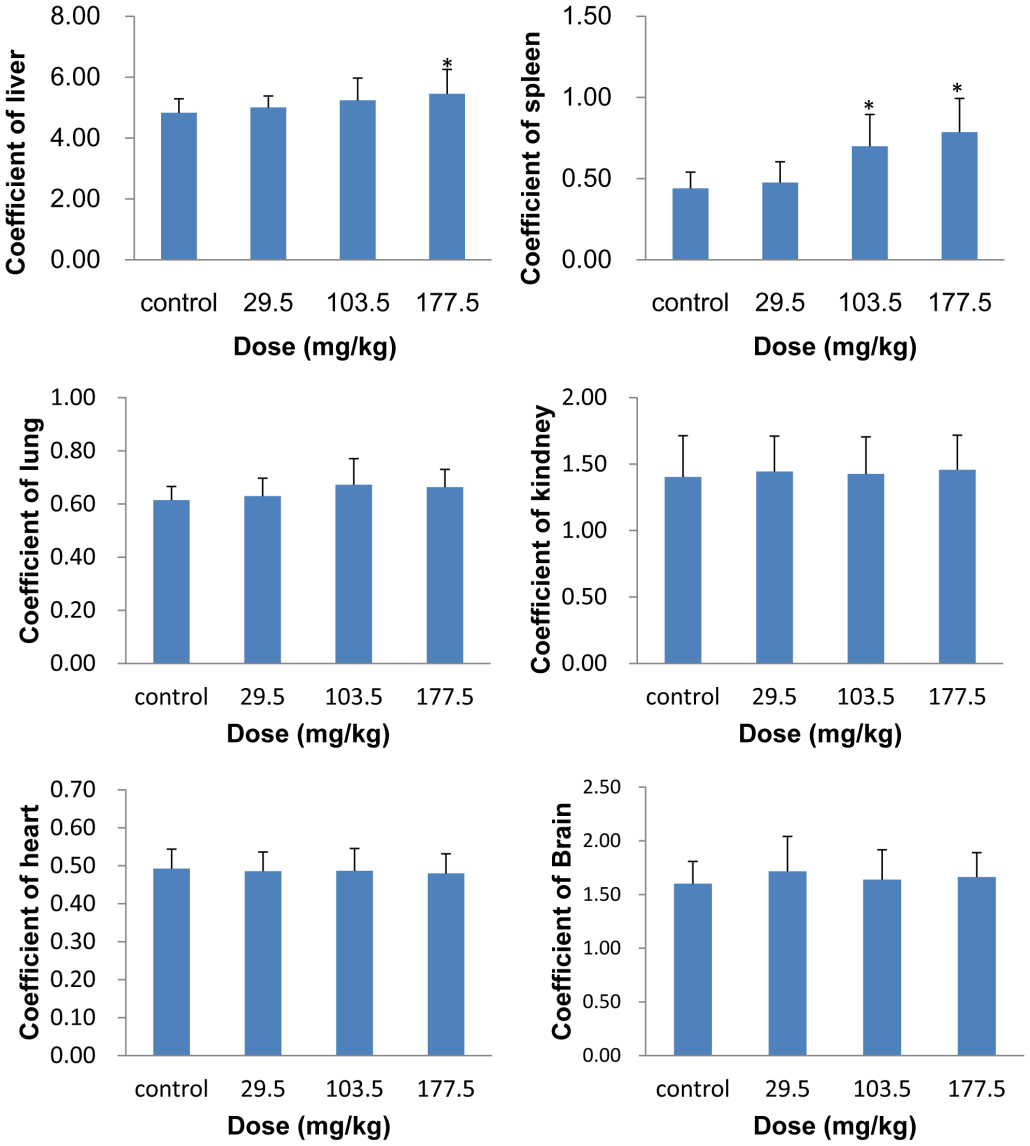
Coefficient of liver and spleen of the mice treated with the SNPs. Data are expressed as mean ± SD (n = 9 or 10). * *p*<0.05 compared with control group using ANOVA.

After the 14 d observation period, the mice were weighed and sacrificed. Major tissues and organs, including the liver, spleen, kidneys, heart, lung and brain were excised and accurately weighed. The weight gain decreased in a dose-dependent manner in the SNP treated mice (Figure 7). No obvious differences were found in the weight gain between the control and the three treated groups (Figure 7). The coefficient of liver was obviously increased in the highest treated group, and the coefficient of spleen was significantly higher in the 103.5 and 177.5 mg/kg treated groups than in the control group (*p*<0.05). The coefficient of lung increased slightly in the three SNP treated groups. No significant difference was observed in the other tissues and organs (Figure 8).

#### Blood biochemical parameters

**Figure 9:**
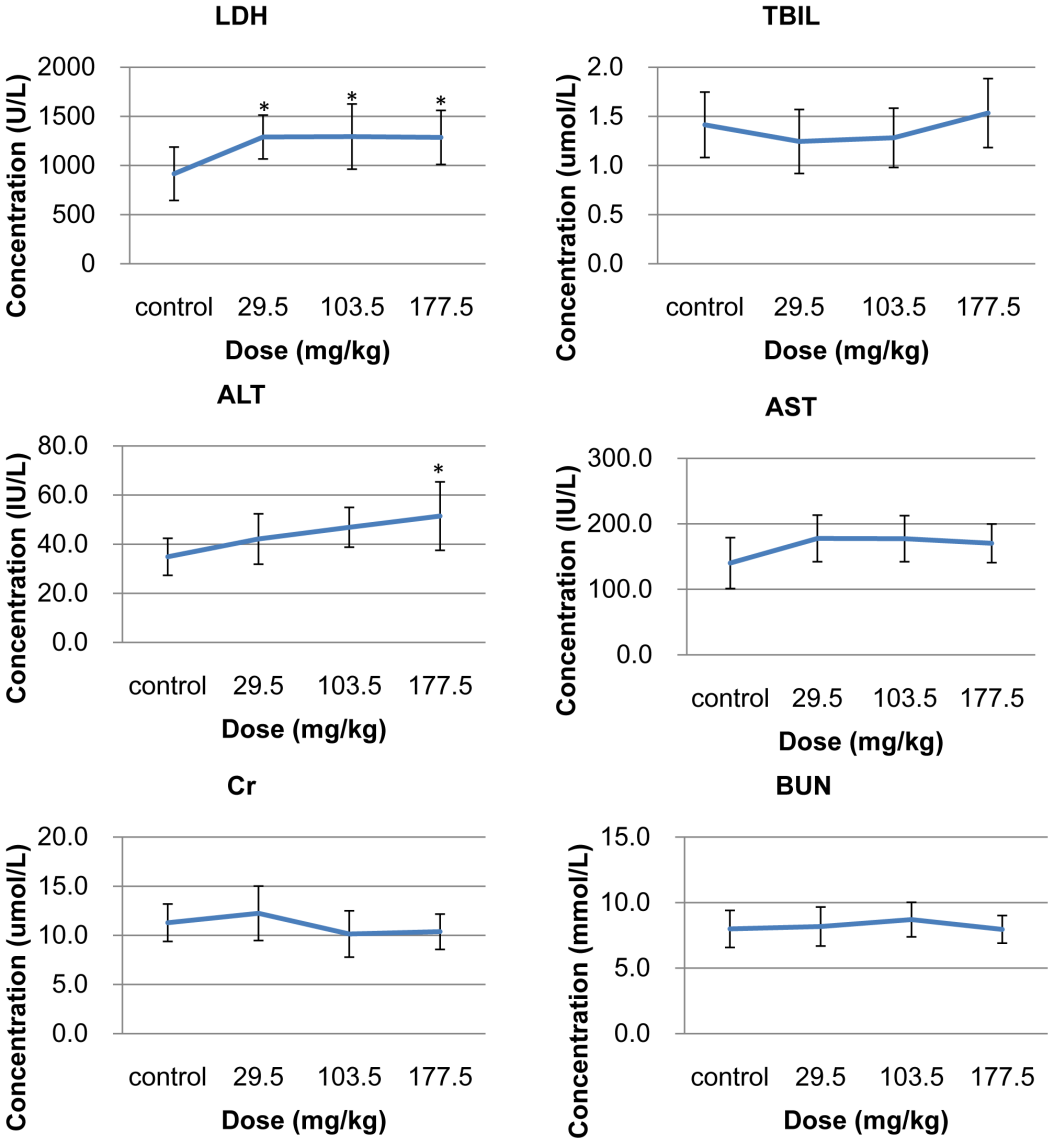
Biochemical analyses in the serum of the mice treated with the SNPs. The SNPs were intravenously administered to mice at 29.5, 103.5 and 177.5 mg/kg. The serum levels of LDH, ALB, AST, ALT, TBIL, BUN and Cr were detected. Data are expressed as mean ± SD (n = 9 or 10). * *p*<0.05 compared with control group using ANOVA.

The results from the blood biochemical examination indicated that the liver could be a target organ for SNPs. The LDH level was significantly higher in the three treated groups than in the control group (*p*<0.05), indicating cell membrane injury and tissue damage in the treated mice. The ALT level was elevated in a dose dependent manner and was significantly higher in the 177.5 mg/kg treated group than in the control group (*p*<0.05). A slight elevation of serum AST was observed in the three SNP treated groups, but there were no obvious changes in serum ALB. No elevation of BUN and Cr, biochemical markers of kidney damage, were observed (Figure 9).

### Pathological examination

**Figure 10:**
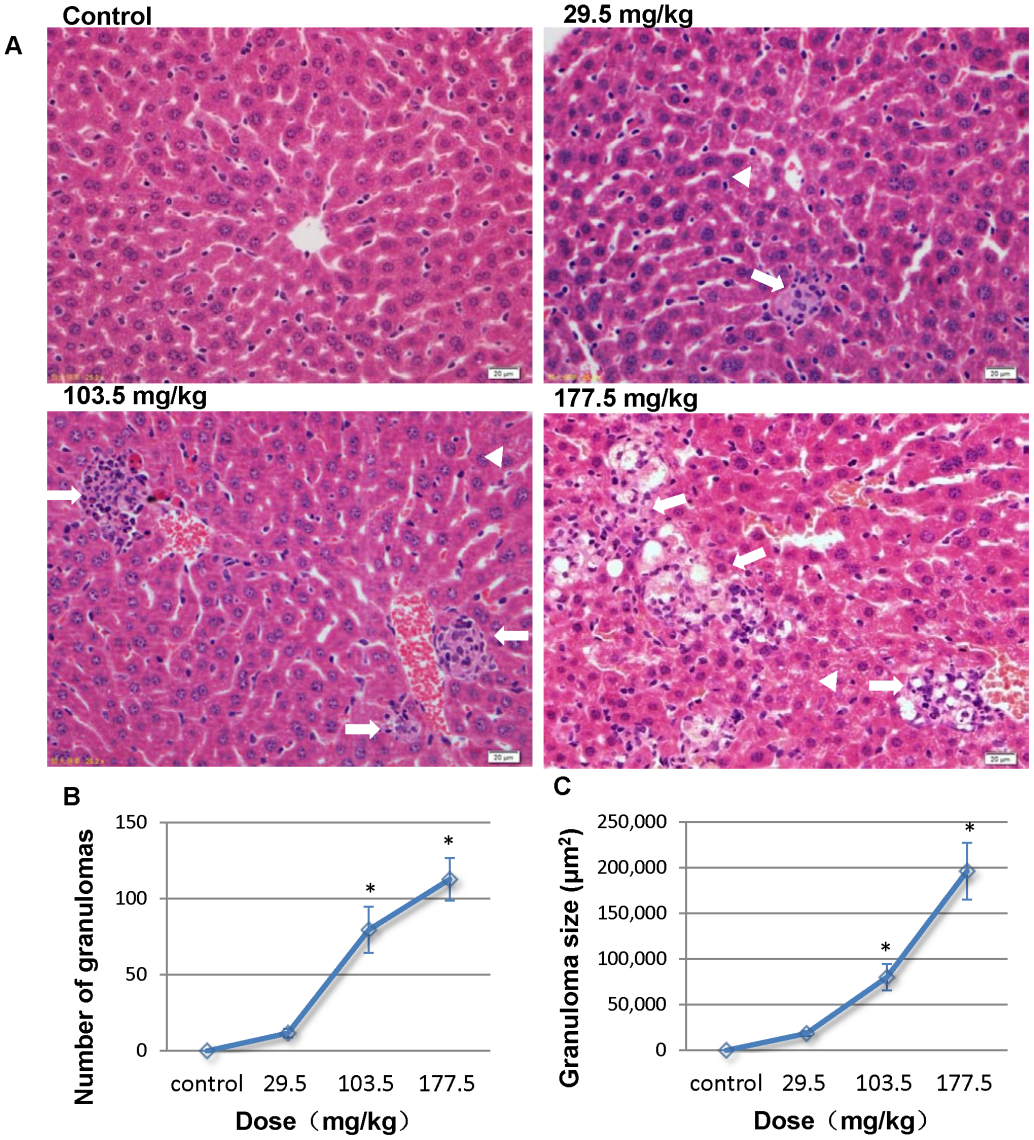
Histological analyses of the liver in the SNP treated mice. (A) Representative liver sections taken from the control mice, 29.5 mg/kg, 103.5 mg/kg and 177.5 mg/kg administered mice at 400× magnification. *White arrows* denote granuloma and lymphocytic infiltration in the liver, and *white triangles* denote hydropic degeneration in hepatocytes. (B) The number of granulomas and (C) the granuloma size in the SNP treated mice increased in a dose-dependent manner in the SNP treated groups. * *p*<0.05 compared with control group using ANOVA. Data are representative of at least eight mice.

**Figure 11:**
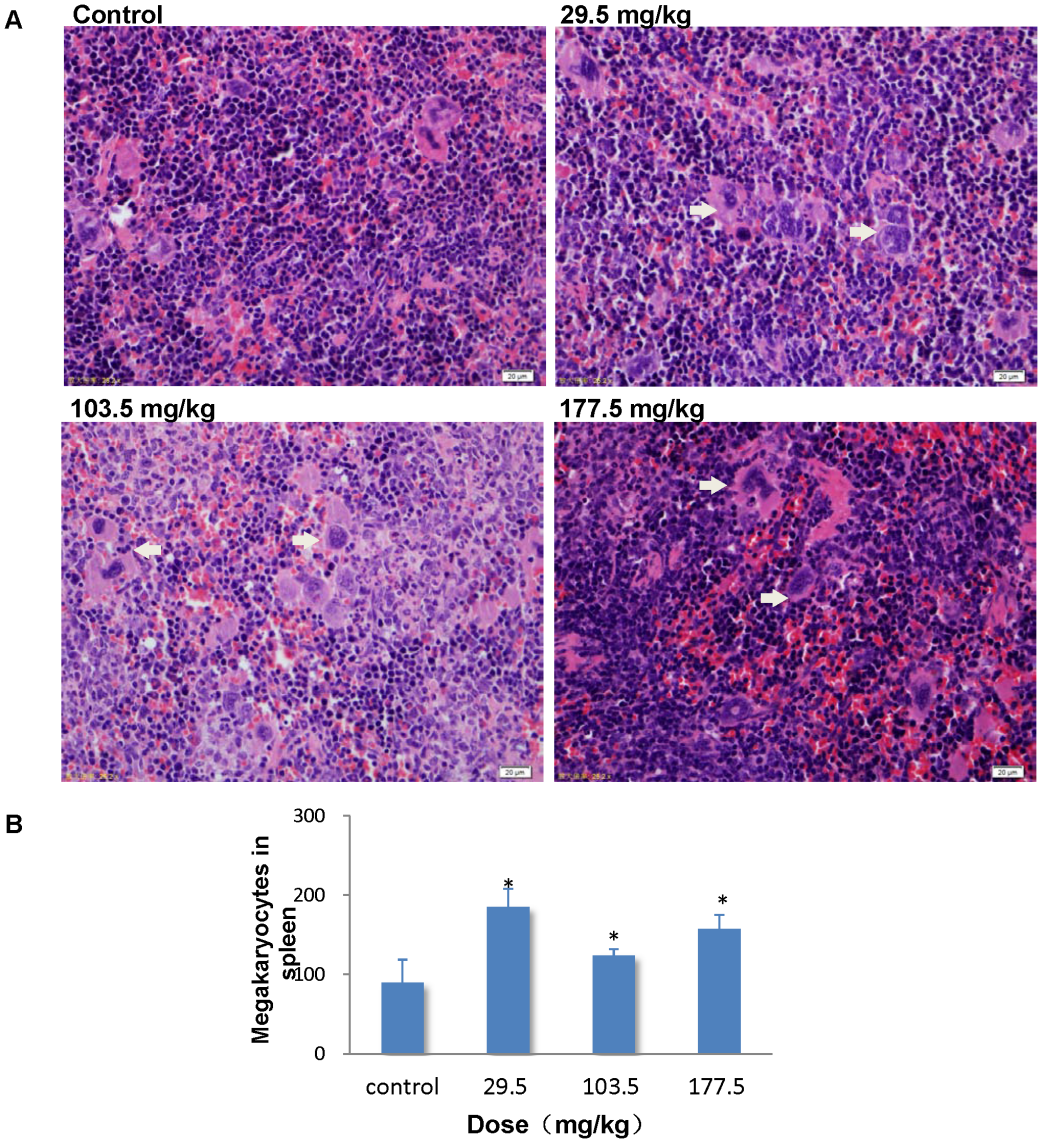
Histological analyses of the spleen in the SNP treated mice. (A) Representative spleen sections taken from the control mice and the 29.5 mg/kg, 103.5 mg/kg and 177.5 mg/kg administered mice at 400× magnification. *White arrows* denote megakaryocytes in the spleen. (B) The number of megakaryocytes in the spleen increased significantly in the SNP treated groups. * *p*<0.05 compared with control group using ANOVA. Data are representative of at least eight mice.

**Figure 12:**
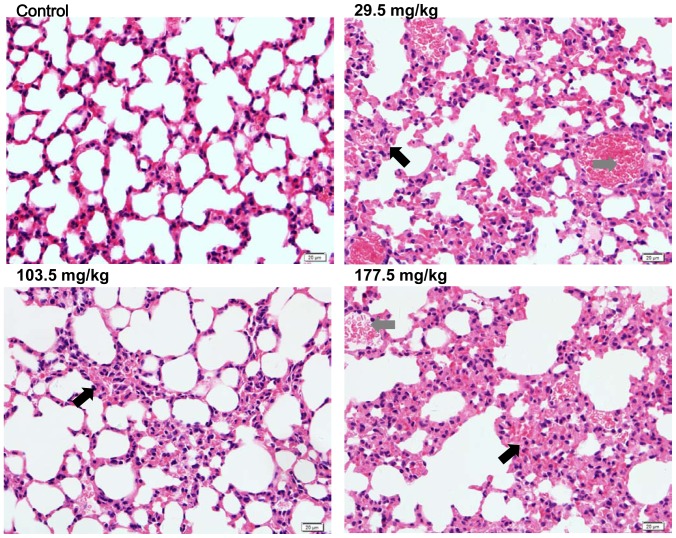
Histological analyses of the lungs in the SNP treated mice. Representative lung sections taken from control mice and 29.5 mg/kg, 103.5 mg/kg and 177.5 mg/kg administered mice at 400× magnification. Images from the SNP treated mice revealed pulmonary interstitial thickening (*black arrows*) and pulmonary arterioles dilatation and congestion (*gray arrows*) in the lung. Data are representative of at least eight mice.

**Figure 13:**
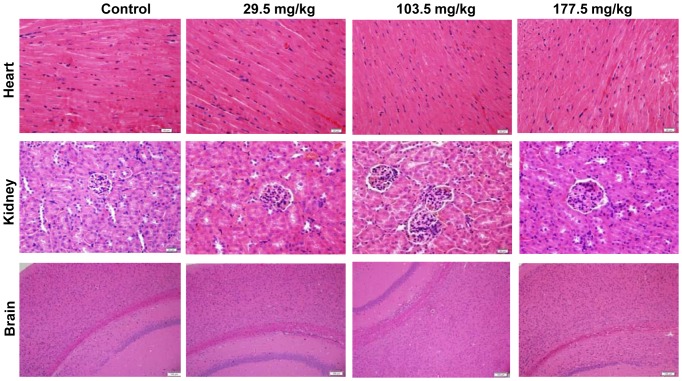
Histological analyses of the heart, kidney and brain in the SNP treated mice. Histological sections were stained with hematoxylin and eosin. Heart, lung and kidney sections were observed under a microscope at 400× magnification, and brain sections were observed at 100× magnification. Data are representative of at least eight mice.

In the control group, liver sections showed normal hepatic cells with complete cytoplasm as well as intact nucleus, nucleolus and central vein (Figure 10A). Lymphocytic infiltration, granuloma formation and hydropic degeneration in the hepatocytes were observed in the livers of the 29.5, 103.5 and 177.5 mg/kg SNP treated mice indicating that the SNPs may be hepatotoxic. Multiple Kupper cells (KCs) were obviously visible disintegrating in the granuloma of the 177.5 mg/kg treated group (Figure 10A). The numbers and sizes of granulomas in the liver increased in a dose-dependent manner in the SNP treated groups (Figure 10B and C). Megakaryocyte hyperplasia was present in the red pulp in the spleen of the SNP treated mice (Figure 11A), and the number of megakaryocytes in the spleen of the SNP treated mice was elevated 0.38 to 1.07 fold more than contol values (Figure11B). Pulmanory hyperemia and pulmonary interstitial thickening were observed in the lungs of the SNP treated mice (Figure 12). The other viscera including the heart, kidney and brain did not show significant changes in morphology in the SNP treated mice (Figure 13).

### Immunohistochemistry stain of CD68

**Figure 14:**
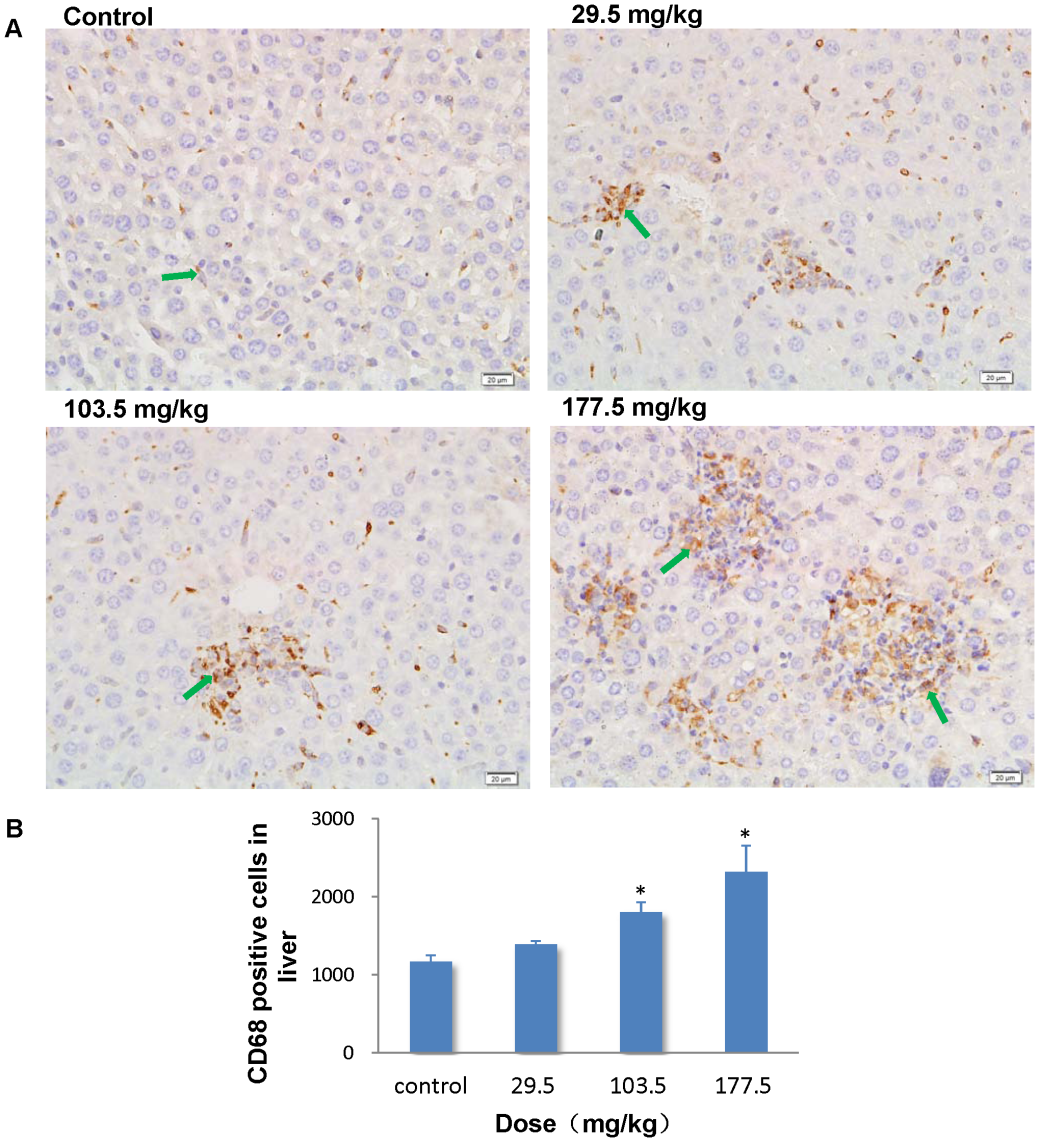
Immunohistochemistry stain of CD68 in the liver. (A) Representative liver sections taken from control mice and 29.5 mg/kg, 103.5 mg/kg and 177.5 mg/kg administered mice at 400× magnification. Hepatic granulomas showed strong positive signals for CD68 in the SNP treated mice. *Arrows* denote CD68 positive cells. (B) The number of CD68 positive cells increased in a dose-dependent manner in the liver from SNP treated mice. * *p*<0.05 compared with control group using ANOVA. Data are representative of at least six mice.

**Figure 15:**
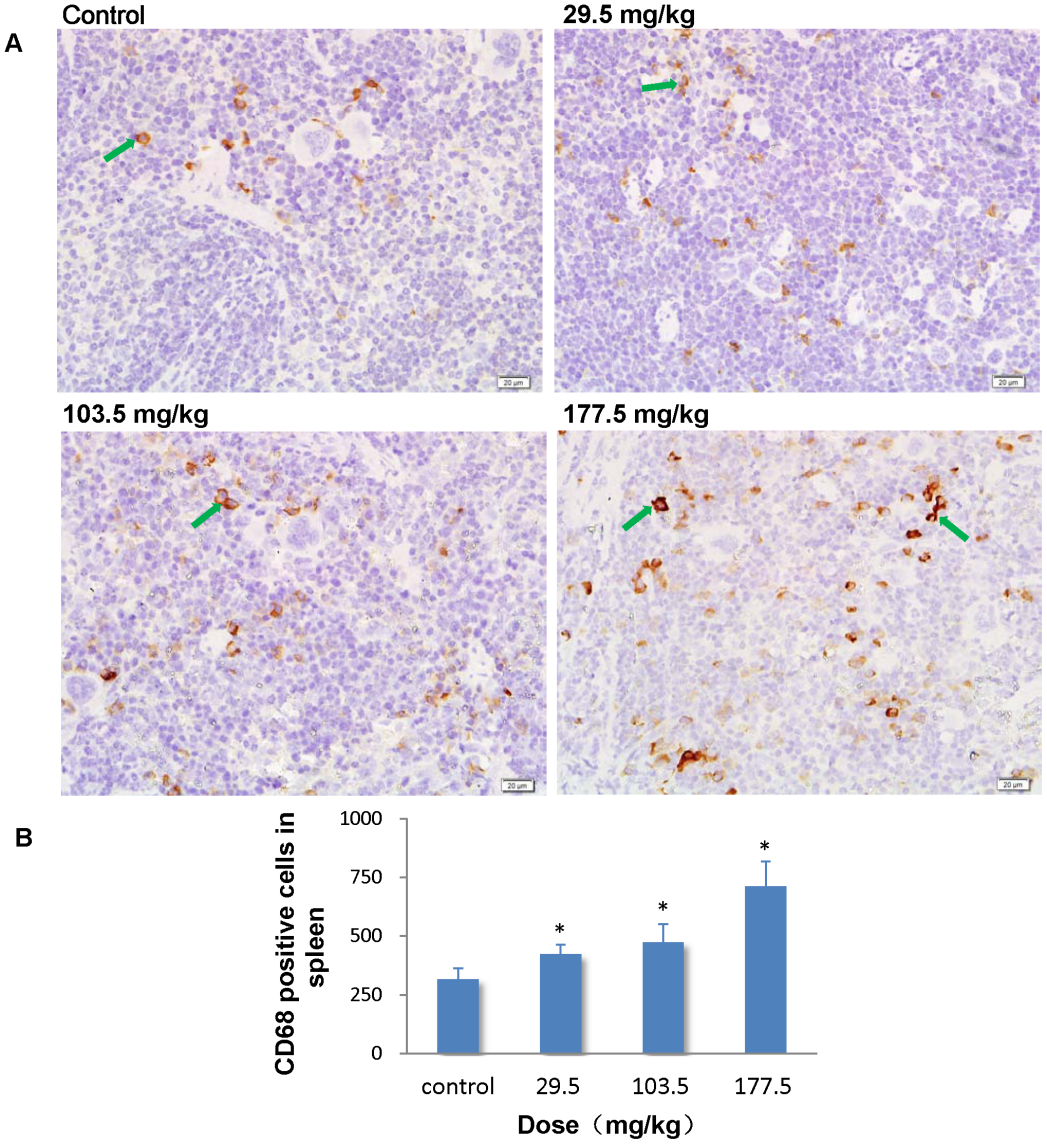
Immunohistochemistry stain of CD68 in the spleen. (A) Representative spleen sections taken from the control mice and the 29.5 mg/kg, 103.5 mg/kg and 177.5 mg/kg administered mice at 400× magnification.CD68 expression increased in the red pulp in the spleen of the SNP treated mice. *Arrows* denote CD68 positive cells. (B) The number of CD68 positive cells increased in a dose-dependent manner in the spleen from SNP treated mice. * *p*<0.05 compared with control group using ANOVA. Data are representative of at least six mice.

**Figure 16:**
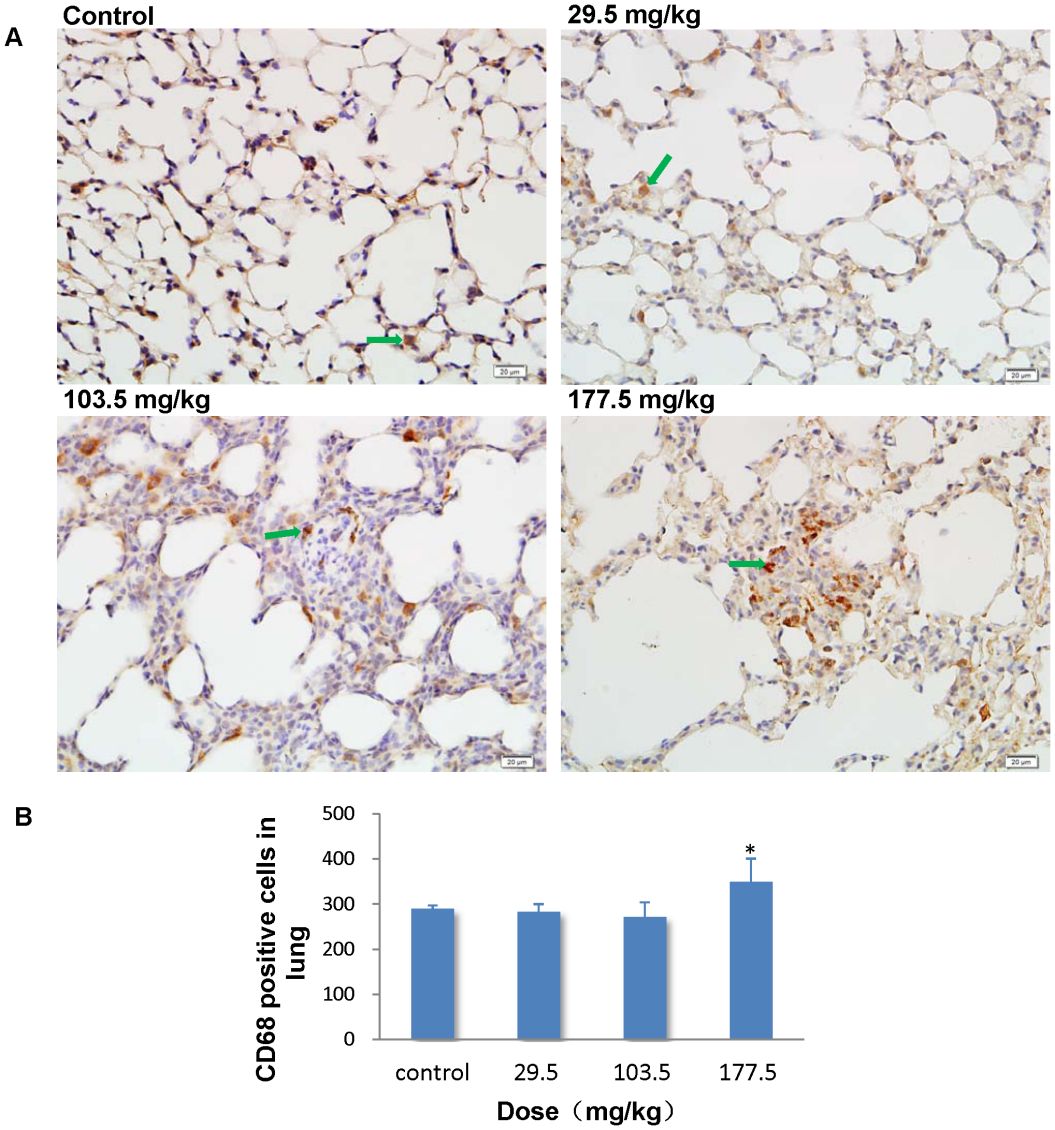
Immunohistochemistry stain of CD68 in the lung. (A) Representative lung sections taken from the control mice and the 29.5 kg, 103.5 mg/kg and 177.5 administered mice at 400× magnification. Positive signals of CD68 expression were occasionally observed in suspected granuloma in the lung sections from the 103.5 and 177.5 mg/kg treated mice. *Arrows* denote CD68 positive cells. (B) The number of CD68 positive cells increased significantly more in the 177.5 mg/kg group than in the control group. * *p*<0.05 compared with control group using ANOVA. Data are representative of at least six mice.

To further investigate the role of macrophages in pathological changes in the liver, spleen and lung, we performed an immunohistochemistry stain of CD68 in the sections of these organs. In the control group, CD68 positive cells mainly located in the hepatic sinusoid in the liver, whereas strong positive signals for CD68 were found in the hepatic granulomas of the SNP treated mice (Figure 14A). Administration of SNPs at 103.5 and 177.5 mg/kg significantly elevated the number of CD68 positive cells in the liver to 1.54 and 1.98 fold more than control values (Figure 14B). The results indicated that the granuloma may result from the fusion of several separated KCs. In the spleen, CD68 positive cells accumulated in the splenic sinus of both the control mice and the SNP treated mice (Figure 15A). But the number of CD68 positive cells in the spleen was significantly greater in the SNP treated mice than in the control mice (Figure 15B). The association between the number of CD68 positive cells in the liver or spleen and SNP exposure was found to be dose-dependent. In the lung, CD68 positive cells mainly localized in the pulmonary interstitial or the alveolar space in the control and 29.5 mg/kg treated mice (Figure 16A). Positive signals for CD68 expression were also observed in suspected granuloma occasionally found in the lung sections of the 103.5 and 177.5 mg/kg treated mice (Figure 16A). But only in the 177.5 mg/kg group did the number of CD68 positive cells increase significantly more than that of the control group (Figure 16B).

### TEM imaging

**Figure 17:**
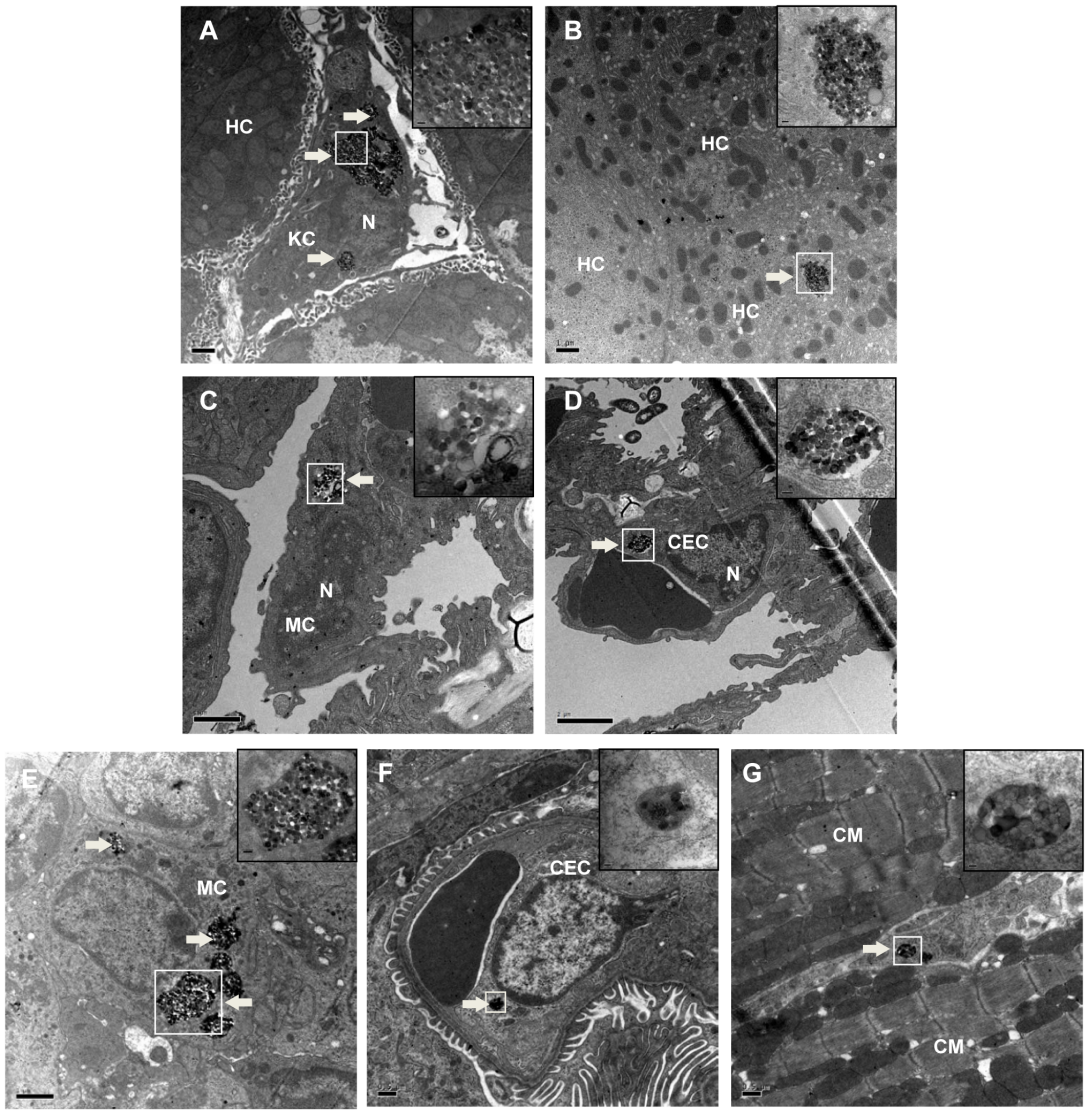
TEM images of the liver, lung, spleen, kidney and heart from the 177.5 mg/kg treated mice. *White arrows* denote phagolysosomes with endocytosed SNPs; the regions in the *white boxes* are magnified on the upper right corner of the same image. (A) SNPs in the Kupffer cell in the liver (5 k), (B) SNPs in the hepacyte in the liver (5 k), (C) SNPs in the macrophage in the lung (10 k), (D) SNPs in the capillary endothelial cell in the lung (6 K), (E) SNPs in the macrophage in the spleen (8 K), (F) SNPs in the capillary endothelial cell in the kidney (8 k), (G) SNPs in the myocardial interstitium in the heart (8 k). N: nucleolus, HC: hepatic cell, MC: macrophage cell, KC: Kupffer cell, CEC: capillary endothelial cell, CM: cardiac myocyte.

In order to confirm the subcellular distribution of SNPs in major tissues, TEM imaging was performed in the 177.5 mg/kg SNP treated mice. SNPs were observed in the resident macrophages in the liver, lung and spleen (Figure 17A, C and E). However, a limited amount of SNPs was found trapped in the hepatocytes in the liver, in the pulmonary capillary endothelial cells in the lung, or in the glomerulus capillary endothelial cells in the kidney (Figure 17B, D and F). The SNPs in the heart were observed only in the myocardial interstitium; none were found in the capillary endothelial cells or in the cardiac myocyte (Figure 17G). The SNPs found in the tissues appeared to aggregate into clumps, enveloped by a lysosomal membrane and without any obvious change of size or shape (Figure 17). No SNPs were found in the TEM samples from the brain.

## Discussion

Because SNPs have become one of the most widely applied nanomaterials in the biomedical and pharmacological fields, an evaluation of their safety is extremely important. In the present study, we estimated the LD_50_ of 64 nm amorphous SNPs through intravenous administration in mice to be 262.45±33.78 mg/kg using the Dixon up-and-down method. Based on the result of the LD_50_, we set a series of doses with equal intervals at 29.5, 103.5, and 177.5 mg/kg to investigate the acute toxicity of SNPs. The results revealed elevated biochemical indexes including LDH, AST and ALT in the SNP treated groups. The histopathology examination indicated lymphocytic infiltration, granuloma formation, and hydropic degeneration in the hepatocytes of the liver. The number of megakaryocytes increased in the spleen and pulmanory hyperemia and interstitial thickening was discovered in the lung by the histological examination. A CD68 immunohistochemistry stain indicated the SNPs induced macrophage proliferation in the liver and spleen.

Our study may contibute to the evaluation of the safety of SNPs in biomedical and pharmacological applications. To date, this is the first study to report the calculated LD_50_ of intravenously administrated amorphous spherical SNPs in mice using a toxicological method. The LD_50_ of 70 nm SNPs administrated by bolus injection in mice has been reported as 45 mg/kg. Possibly, a rapid bolus injection, which is commonly used in medical imaging, may make the 70 nm SNPs more toxic. In addition, the different strains of mouse models and the difference in LD_50_ evaluation methods could also contribute to the different values of LD_50_ obtained in these two studies. [22]. Traditional LD_50_ determinations use multiple animals at 5–7 defined dose intervals; however, the Dixon up-and-down method used in our study reduces the number of animals required to estimate LD_50_ values. During the procedure, the intravenous injection of the SNPs at 325.5 mg/kg was often lethal (Figure 3). The histopathology examination afterwards revealed serious hepatic necrosis, pulmonary microthrombi, hyperemia and haemorrhage in the main organs of the dead mice. An early study by Movat et al. demonstrated that nanoparticles could lead to platelet activation and aggregation [23]. Recently, Nabeshi et al. reported that the interaction between the SNPs and the instinct coagulation factors XII induced platelet depletion and consumptive coagulopathy after systemic exposure [22]. Coincidentally, we observed progressive tail ischemic necrosis in the 177.5 mg/kg group of this subsequent study, which might be ascribed to the microthrombi formation in the local blood vessels induced by the SNPs. Thus, we hypothesized that when a large amount of SNPs entered the bloodstream they might induce platelet activation and aggregation and then disseminate intravascular coagulation (DIC), followed by wide microthrombus formation, multiple organ failure and death. Fibrin staining of the lung sections in the dead mice with MSB proved fibrin thrombi formation in the pulmonary arterioles in the lungs of the dead mice which indicated that the SNPs induced DIC and then death in the dead mice. The results of our study may provide some evidence that the thrombogenicity of the SNPs should be taken into serious consideration in the biological and pharmacological application of SNPs.

The subsequent biodistribution analysis of SNPs in the 177.5 mg/kg group revealed that SNPs mainly distributed in liver (10.24%ID/g), spleen (34.78%ID/g), and lungs (1.96%ID/g). Most of the SNPs accumulated in the resident macrophages of these organs, while only a small amount distributed in the hepatocytes of the liver or in the capillary endothelial cells of the lungs and kidneys. It has been reported that the bioretention of SNPs could last more than 4 weeks after an intravenous injection [18]. However, in our study, almost 47.45% of the injected SNPs were distributed in the liver and spleen 14 days after injection, which may be attributed to the powerful scavenger and defense function for blood borne xenobiotics from MPS in these organs, which has been demonstrated by TEM imaging and CD68 immunohistochemistry stain. The results of the immunohitochemistry stain indicated that the SNPs can induce macrophage activation and proliferation in the liver and spleen, thus leading to the injury in these organs.

One of the most important histological observations in the liver was the presence of hepatic granuloma formation. Granuloma formation initiates from phagocytosis of the macrophage. The macrophage engulfs xenobiotics such as SNPs, which is followed by inflammatory cytokines and the release of chemokines and results in oxidative stress and cell damage as reported by Park et al. both in peritoneal macrophage and the RAW264.7 cell line [24]. In the liver, when SNPs are phagocytized by KCs, they may induce KC damage and disintegration, which is then released and taken up by other KCs. The ultimate end to this recurring reaction, with parallel lymphocytes and neutrophils recruited by the cytokines and chemokines released by KCs, is the formation of granuloma [25]. In essence, granuloma formation is an innate immune response designed to isolate xenobiotics, foreign body granuloma [26]. Granuloma induced by nanomaterials through different routes including intratracheal instillation, and intraperitoneal and intravenous injection have been reported by several research teams [17], [19], [22], [26], [27], [28], [29]. In the early phase, the granuloma consists of different kinds of cells such as macrophages, lymphocytes, and neutrophils, but in the late phase, the granuloma turn into fibrosis, which has been observed in our on-going work. Fibrosis granuloma in the liver is caused by repeated intravenous administration of SNPs and occurs for a longer period; research into the mechanism is under way.

As the main filter and store of blood, it follows that the spleen becomes the largest holder of SNPs. TEM imaging and the CD68 immunohistochemistry stain revealed that marophages played an important role in the retention of SNPs in the spleen. In our study, histological hyperplasia of megakaryocytes in the red pulp of the spleen was observed in SNP treated mice, indicating splenic extramedullary hematopoiesis, which is associated with pathological conditions [30], [31], [32], [33]. Kwon et al. demonstrated that inhaled fluorescent magnetic nanoparticles induced extramedullary hematopoiesis in the spleen of mice without pulmonary abnormalities [34]. However, the mechanism behind nanoparticle induced extramedullary hematopoiesis is still not clear. The long retention of SNPs in the spleen may impact the splenic immune function. Park et al. demonstrated that SNPs decreased splenic cell viability, and induced phenotypic alterations of lymphocytes derived from the spleen after intraperitoneal injection [24]. Taken together, the toxicity and the mechanism induced by SNPs in the hemopoietic system and the immune system need to be clarified. A group of colleagues from our team are working on the related research.

In our study, we also observed changes in several biochemical indexes. An elevated serum LDH in the SNP treated groups indicated cell membrane destruction in the main organs, which was probably induced by the distribution and retention of SNPs in the relevant cells of the main organs, as observed by TEM imaging. The serum AST and ALT levels were also raised in the SNP treated groups. This elevation illustrates the hepatocyte injuries which correlate with the histological changes of hydropic degeneration in the hepatocytes of the liver. The injuries may be attributed to two causes. First, the SNPs may induce KC released cytokines such as TNF-α which could in turn induce apoptosis and necrosis of the neighboring hepatocytes [35]. Or possibly the cell injury may be caused by phagocytosis of the SNPs by the hepatocytes themselves as we observed by TEM imaging. This has also been demonstrated in SNP treated human hepatic cell line L-02 in vivo (data not published). The levels of Cr and BUN in the SNP treated groups remained normal intimating that SNP injection did not affect kidney function.

This experiment was designed as a pilot study for our further research. For the first time the LD_50_ of 64 nm amorphous SNPs was estimated as 262.45±33.78 mg/kg using the Dix up-and-down method. The subsequent gross anatomical and histological examination of the main organs of dead animals revealed that SNPs probably induced DIC related to death. So, we would like to emphasize the importance of thrombogenicity of SNPs during the application and safety evaluation. In the subsequent acute toxicity and biodistribution study, we found that during short-term systemic exposure, SNPs mainly distributed in the liver, spleen and lung and induced injury in these organs. MPS played important roles in the distribution and accumulation of SNPs as well as in the formation of tissue lesions. Further studies on the roles of MPS and the related mechanism would provide useful information for the evaluation of the safety and biological application of SNPs.
